# Pancreas of coxsackievirus-infected dams and their challenged pups: A complex issue

**DOI:** 10.1080/21505594.2019.1589364

**Published:** 2019-03-22

**Authors:** Sona Sarmirova, Maria Borsanyiova, Brigita Benkoova, Michaela Pospisilova, Rajkumar Arumugam, Katarina Berakova, Pavol Gomolcak, Jay Reddy, Shubhada Bopegamage

**Affiliations:** a Enterovirus Laboratory, Institute of Microbiology, Faculty of Medicine, Slovak Medical University, Bratislava, Slovak Republic; b School of Veterinary Medicine and Biomedical Sciences, University of Nebraska-Lincoln, Lincoln, NE, USA; c Martinske biopticke centrum s.r.o, Zilina, Slovak Republic; d Immunohistochemical Laboratory, Medical Laboratory Pathology and Cytology, Cytopathos, s.r.o, Bratislava, Slovak Republic

**Keywords:** Coxsackievirus B4-E2, dams, pancreas, mice, gravidity, offspring

## Abstract

Enteroviral infections are frequent, often asymptomatic in humans and during gravidity.

The present study is an extension of our previous investigations where we had shown pancreatitis in challenged pups of CVB4-E2-infected dams. Present investigation describes the effect of gestational infection with this virus on the pancreas of both dams and their challenged pups. Gravid CD1 outbred mice were orally infected with CVB4-E2 virus at different gestation times. Pups were challenged orally with the same virus after 25 days of birth. Organs were collected at selected intervals postinfection (p.i.), and replicating virus and viral-RNA copies were analyzed. Additional readouts included histopathology and immunohistochemical (IHC) analysis for localization and identification of Ly6G+ cells (neutrophils), CD11b+ cells (macrophages), and viral protein in pancreatic tissue sections of the infected dams and their challenged pups. Our results show the presence of replicating virus in the pancreas of infected dams and their challenged pups, with inflammation leading to chronic necrotizing pancreatitis and atrophy of pancreatic acini of the dams and their offspring. IHC analysis of the infiltrating cells showed pronounced Ly6G+ neutrophils in dams only, whereas CD11b+ macrophages were present in tissues of both, the pups and the dams. Time of infection during gravidity as well as the p.i. intervals when mice were sacrificed influenced the pancreatic pathophysiology in both groups. We conclude that coxsackievirus infection during pregnancy is a risk factor for chronic affliction of the exocrine tissue and could affect endocrine pancreas in the mother and child.

## Introduction

A wide range of clinical manifestations are associated with enterovirus (EV) infections. In general, these viral infections are mostly subclinical and may go unnoticed. Genus *Enterovirus* of the family *Picornaviridae* contains a large number of human viral pathogens, distributed in seven species *Enterovirus A* to *D* and *Rhinovirus A* to *C* (https://talk.ictvonline.org/ictv-reports/ictv_online_report/positive-sense-rna-viruses/picornavirales/w/picornaviridae) [1].

Transmission of EVs from a mother to the fetus and newborn may be antenatal, perinatal, at the time of delivery, or postnatal [2,3]. Existence of several case reports suggest that EV infections during pregnancy may cause meningitis, preterm delivery, neonatal myocarditis, fetal growth retardation, or even embryopathy [4–21]. However, intrauterine transmission of EVs is nevertheless discussed with ambiguity. Seroepidemiological studies associate coxsackievirus B4 infection during pregnancy with childhood onset of type 1 diabetes (T1D) [22–25]. Although EV infections are common, they are not included in the routine clinical screening tests during pregnancy. Experimental studies related to the influence of enteroviral infections on the course of gravidity are limited [26–34].

Our previous work [26] showed severe inflammation of the pancreatic acinar tissue in homologously challenged progeny of dams infected during gestation at different time intervals. However, the study was limited to only a single time point observation, namely day 5 postinfection (p.i.) after challenging the litter of these infected dams. The organs of the dams were not investigated. The objective of our present study therefore was to systematically follow up firstly the maternal infection during pregnancy (day 3 to day 14 p.i.) and a subsequent homologous infection of the offspring (day 5 to day 21 p.i.) over a longer period than our previous study [26], with a focus on the pancreas.

## Results

Observations at later intervals could not be made in the dams which were infected late during gravidity as mice delivered on time and nursed the suckling and therefore could not be sacrificed.

## Replicating virus in selected organs of experimentally infected dams

Spread of replicating virus was confirmed in most of the collected organs after three blind passages (P1, P2, P3). 75% (3/4) placentas of dams infected in the first week (day 4/E.4) showed replicating virus by the third blind passage P3 when studied at days 3, 5 and 14 p.i. All placentas were positive by P3 in dams infected in the second week (day 10/E.10) at day 5 p.i., and at day 3 p.i., only 50% of placentas were positive in this group and in the dams infected at day 17/E.17.

Presence of replicating virus was observed in the umbilical cords in 50% (2/4) of dams (infected in second week) when checked at day 3 p.i. All umbilical cords showed replicating virus (dams infected in the first week and third week) (Table 1) and at day 5 p.i. of the dams infected in the second week.

**Table 1.** Presence of replicating virus in different organs of the gravid mice.

	Number of positives/total number of mice at different blind passage (P*) levels on selected days after CVB4-E2 infection								
Organs of gravid mice infected at different time points	Day 3 p.i.**/E.7***			Day 5 p.i./E.9			Day 14 p.i./E.18		
Week 1 Day 4/E.4	P1	P2	P3	P1	P2	P3	P1	P2	P3
Heart	4/4	4/4	4/4	3/4	4/4	4/4	2/4	4/4	4/4
Pancreas	4/4	4/4	4/4	4/4	4/4	4/4	3/4	4/4	4/4
Umbilical cord	3/4	4/4	4/4	3/4	4/4	4/4	2/4	4/4	4/4
Placenta	3/4	3/4	3/4	2/4	2/4	3/4	0/4	1/4	3/4
Week 2 Day 10/E.10	Day 3 p.i./E.13			Day 5 p.i./E.15					
Heart	3/4	4/4	4/4	4/4	4/4	4/4	–	–	–
Pancreas	3/4	4/4	4/4	4/4	4/4	4/4	–	–	–
Umbilical cord	2/4	2/4	2/4	4/4	4/4	4/4	–	–	–
Placenta	2/4	2/4	2/4	4/4	4/4	4/4	–	–	–
Week 3 Day 17/E.17	Day 3 p.i./E.20								
Heart	2/4	4/4	4/4	–	–	–	–	–	–
Pancreas	2/4	4/4	4/4	–	–	–	–	–	–
Umbilical cord	3/4	4/4	4/4	–	–	–	–	–	–
Placenta	2/4	2/4	2/4	–	–	–	–	–	–

P* = passage level. p.i.** =postinfection. E.7*** =embryonic age. – = delivered in term.

All heart and pancreatic tissues of infected dams showed a replicating virus after 3 blind passages. Mice infected in the third week of gravidity were not studied longer than day 3 p.i. as they delivered in a term (day 21).

## RNA copies in the pancreas of dams infected at different weeks of pregnancy and their challenged pups

### Gravid dams

**Figure 1.**
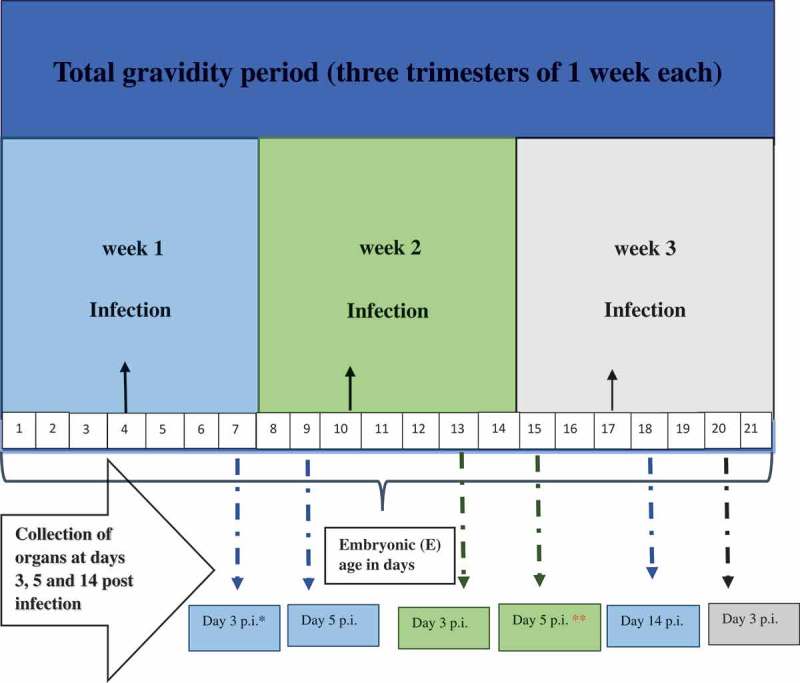
Schematic diagram: Gestational (G) period, embryonic (E) age, infection timings, and organ collection schedules.

**Figure 2.**
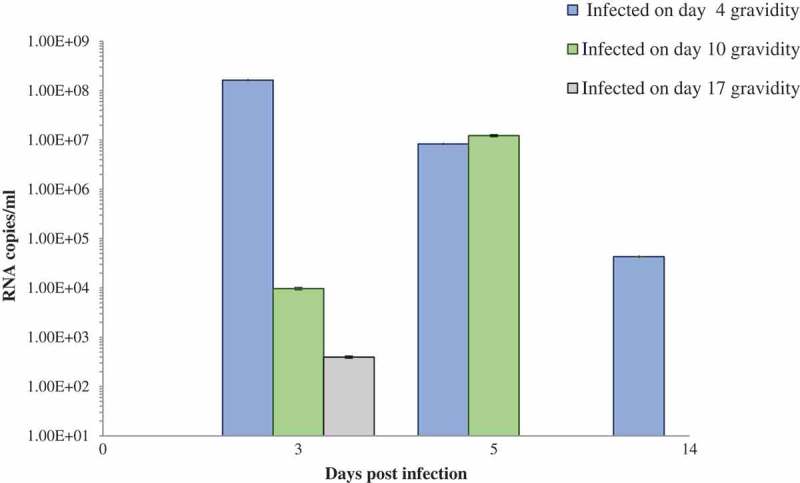
Viral RNA load in pancreas of dams infected during gravidity.

Considering our published observations of infiltration of the acinar tissue in the litter of previously infected dams [26], we have focused our investigations on the pancreas. On day 3 p.i. (E.7, E.13, E.20 as in Figure 1), viral RNA copies in the pancreas of dams infected in the first week were highest as compared to the pancreas of mice infected in the second week of gravidity (*p* < 0.05; *p* = 0.0007) and third week (*p* < 0.05; *p* = 0.0007) as shown in the graph (Figure 2). The results also demonstrate that in the pancreas of dams infected in the second week, viral RNA load was higher than in the pancreas of dams infected in the third week of gravidity (*p* < 0.05; *p* = 0.04) at day 3 p.i. (E.13 and E.20).

The viral RNA copies were significantly lower on day 5 p.i. (E.9) than on day 3 p.i (E.7) in the pancreas of gravid mice infected in the first week of infection (*p* < 0.05; *p* = 0.0008). On the other hand in the mice infected in the second week of gravidity, the copies were increased on day 5 p.i. (E.15) as compared to day 3 p.i. (E.13) (Figure 2).

### Challenged pups of infected dams

**Figure 3.**
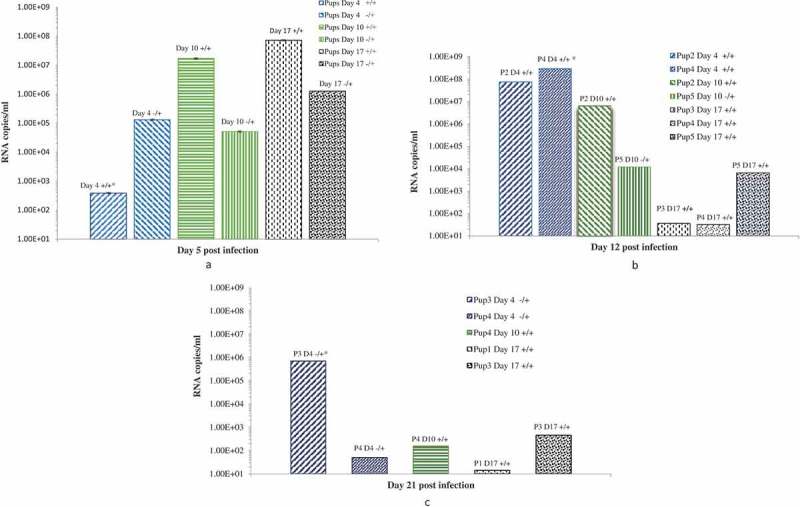
(a) Viral RNA load in pancreas of the challenged pups of CVB4-E2-infected and control dams. (b) Viral RNA load in pancreas of “individual” challenged pup showing positivity at day 12 postinfection of CVB4-E2-infected and control dams. (c) Viral RNA load in pancreas of “individual” challenged pups showing positivity at day 21 postinfection of CVB4-E2-infected and control dams. *+/+ both mother and pup infected; −/+ mother mock-infected and pup-infected, P = pup; 1, 2, 3, 4, 5 number code of individual pup for that catergory. Days 4, 10, and 17 show the time of infection of the dam.

Highest viral RNA copies were in the pancreas of the pups (+/+) of previously infected dams as compared to the group of pups (−/+) of uninfected dams on day 5 p.i. (Figure 3(a)). However, at this time point, a statistically significant difference was observed between the +/+ and – /+ pups of dams infected on day 10 (E.10) of gravidity (*p* < 0.05, *p* = 0.0103).

On day 12 p.i. as seen in Figure 3(b), only a few mice (mainly the +/+ pups) showed the presence of viral RNA, with lower copies in the heart as compared to the pancreas (heart data not shown). On day 21 p.i. similarly, lower copies of RNA were observed only in the pancreas of few pups, belonging to groups +/+ and –/+ (Figure 3(b,c)).

Pups +/– did not show the presence of either replicating virus or viral RNA by quantitative real-time RT-PCR and was therefore not carried out in these pups.

## Histopathological observations in the pancreatic tissues of dams

**Figure 4.**
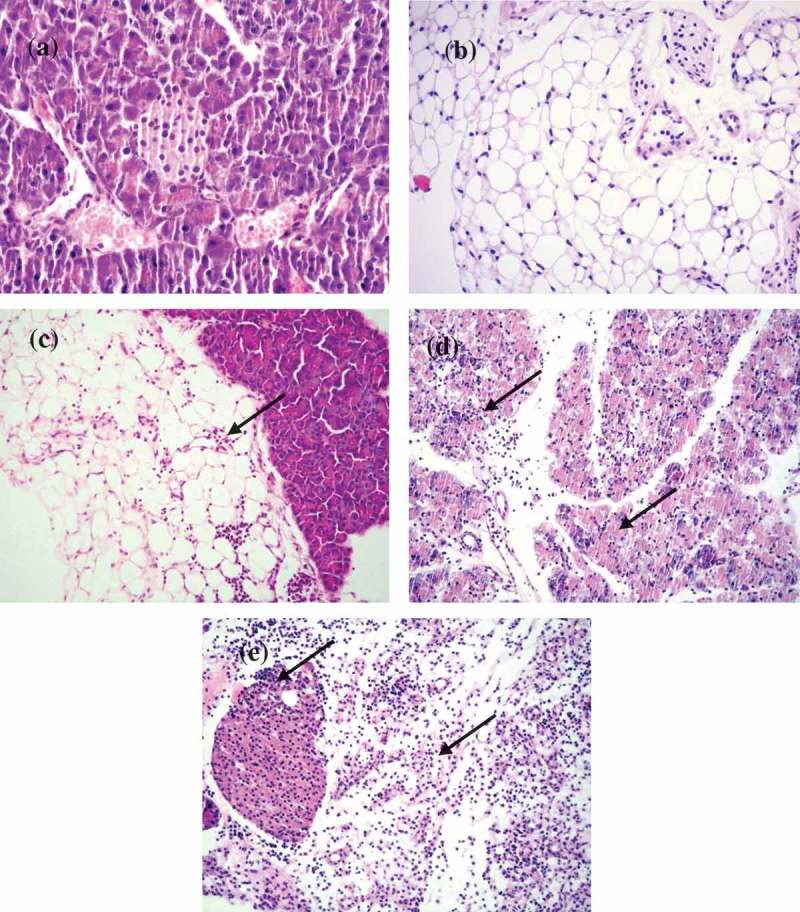
**Histopathological changes in pancreas tissues of CVB4-E2 infected and control dams**. (a) Absence of changes in the acinar and endocrine pancreas tissue (20×) and in the peripancreatic fat tissue (20×) (b) of a dam mock infected on day 4/E.4 sacrificed on day 3 p.i./E.7. (c) Focal infiltration in the peripancreatic fat tissue of virus-infected dam on day 10/E.10, sacrificed on day 3/E.13 p.i. (20×). (d) Pancreatitis in the acinar tissue showing inflammatory phase in the tissue of dam infected with the virus on day 4/E.4 sacrificed on day 3 p.i./E.7 (20×). (e) A stage of transition from acute to a subacute condition with reparative changes, lymphoplasmacytic inflammation with several incipient capillaries, and formation of inflammatory granulation in the defunct parenchymal pancreatic tissue. The lymphocytic infiltrates were also observed in the pancreatic islets of dams infected with the virus on day 10/E.10 (20×) and sacrificed on day 5 p.i./E.15.

The pancreas of the mock-infected dams were without any histopathological changes in the acinar, endocrine, and peripancreatic tissues (Figure 4(a,b)). Table 2 shows the infiltration grades in the pancreas of the infected mice at different time points. Dams infected on day 4/E.4 of gestation show focal interstitial infiltrates with lymphocytes in the peripancreatic fat tissue and in the acinar tissue on day 3 p.i./E.7 (Figure 4(c,d)). We did not observe any infiltration in the islets (endocrine pancreatic tissue) at this time point. The acinar infiltration increased intensively on day 5 p.i./E.9. On day 14 p.i./E.18, the exocrine (acinar) pancreas had recovered and the tissues were normal without changes.

**Table 2.** Grades of destruction in the pancreas tissues of CVB4-E2 infected dams.

Pancreatic tissue of gravid mice infected at different time points	Infiltration status in the pancreas at different days post-CVB4-E2 infection					
	Number of positives/ total	Grade	Number of positives/total	Grade	Number of positives/ total	Grade
Week 1 Day 4/E.4	D3 p.i.*/E.7**		Day 5 p.i./E.9		Day 14 p.i./E.18	
Peripancreatic fat	2/4	+1	4/4	+1	0/4	N
Acinar	2/4	+1 to +2	2/4	+3 to +4	0/4	N
Endocrine	0/4	None	0/4	None	0/4	N
Week 2 Day/E.10	Day 3 p.i./E.13		Day 5 p.i./E.15			
Peripancreatic fat	2/4	+1	3/4	+1	NA	
Acinar	2/4	+1	4/4	+1 to +4		
Endocrine	3/4	+1	4/4	+2 to +4		
Week 3 Day/E.17	Day 3 p.i./E.20					
Peripancreatic fat	0/4	N	NA		NA	
Acinar	0/4	N				
Endocrine	0/4	N				

p.i.** =postinfection. E.7*** =embryonic age. N = no infiltration. NA = not applicable – mice delivered in term.

In the pancreas of dams infected on day 10/E.10, we observed little infiltration of the peripancreatic fat and the acinar tissues on day 3 p.i./E.13. The acinar tissue infiltration increased intensively on day 5 p.i./E.15 denoting a stage of transition from the acute inflammatory phase to a subacute condition with reparative changes, where lymphoplasmacytic inflammation was already prevalent, and numerous incipient capillaries were present, with the formation of inflammatory granulation in the defunct parenchymal pancreatic tissue. The lymphocytic infiltrates were seen in the pancreatic islets (Figure 4(e)). Infiltrates were not observed in the pancreatic tissue of dams infected on day 17/E.17 of gestation on day 3 p.i./E.20.

## Histopathological observations in pancreas tissues of challenged pups of the infected dams

**Figure 5.**
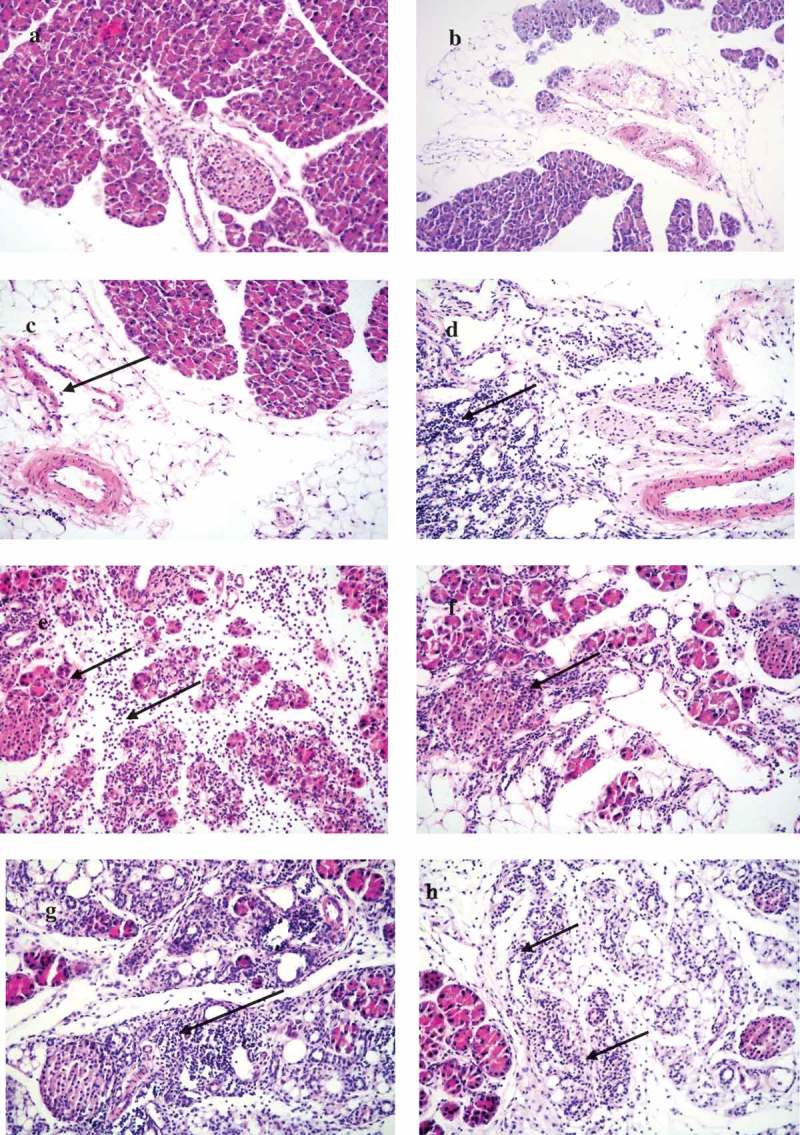
Histopathological changes in the pancreatic tissues of the virus challenged pups of CVB4-E2 infected and pups of mock control dams. (a) Absence of changes in the acinar and endocrine pancreatic tissue (20×) dam infected on day 4/E.4 of gestation and virus challenged pup sacrificed on day 5. (b) Peripancreatic fat tissue (20×) of a mock-infected dam on day 4/E.4 of gestation and (−/−) pup mock challenged and sacrificed on day 5 p.i. (c) Mild and severe (d) forms of infiltration in the peripancreatic fat tissue of a virus challenged two different pups (−/+) of a dam mock-infected on day 4/E.4 of gravidity (20×), pups sacrificed on day 5 p.i. (20×). (e) Chronic pancreatitis and atrophy of the acinar tissue of a challenged pup (+/+) sacrificed on day 5 p.i. born to dam infected with virus on day 10/E.10 of gravidity, (f) chronic pancreatitis and atrophy of the acinar tissue and infiltration in the endocrine islet of a challenged pup (+/+) sacrificed on day 21 p.i. (20×) born to dam infected with the virus on day 10/E.10 (20×) in the tissue of pup sacrificed on day 21 p.i., (g) chronic pancreatitis in the acinar tissue of the virus challenged pups (+/+) of dam infected on day 17/E.17 of gestation day, pup sacrificed on day 12 p.i., absence of infiltration of the islets (20×), (h) chronic pancreatitis and atrophy in another challenged pup (+/+) sacrificed on day 12 p.i. (20×) of dams infected on day 17/E.17 of gestation day.

Our focus is on the pancreas of challenged pups considering the fact that histopathological changes (infiltration) in heart were mild only on day 5p.i. in the +/+ pups and were absent in −/− pups as observed in our previous studies [26]. Tables 3–5 show histopathological changes of the pancreatic tissue with the number of affected challenged pups. Each table represents a group of challenged pups with a maternal infection at a particular time during gestation: day 4/E.4, day 10/E.10, and day 17/E.17. Designation used for the groups of pups of the infected or mock-infect dams were: pups challenged with the virus were +/+ and −/+; pubs challenge with PBS (mock-infected) +/− and −/− . In Tables 3–5 records the grades of histopathological changes. Figure 5(a–f) show the actual histopathological changes.

**Table 3.** Histopathology of pancreas tissues of challenged pups of dams infected in the first week of gestation (day 4/E.4).

Pancreatic tissue of pups	Infiltration status in the pancreas at different days post-CVB4-E2 or mock infection					
	Number of positives/ total	Grade	Number of positives/total	Grade	Number of positives/ total	Grade
	Day 5 p.i. *		Day 12 p.i.		Day 21 p.i.	
+/+**						
Peripancreatic fat	0/5	None	1/5	+2	3/5	+1
Acinar	2/5	+1 to +2	2/5	+4	0/5	None
Endocrine	0/4	0	0/5	None	0/5	None
****−**/+**						
Peripancreatic fat	4/5	+1 to +3	0/5	None	0/5	None
Acinar	0/5	None	0/5	None	0/5	None
Endocrine	0/5	None	0/5	None	0/5	None
**+/−**						
Peripancreatic fat	0/5	None	2/5	+1	3/5	+1
Acinar	0/5	None	0/5	None	0/5	None
Endocrine	0/5	None	0/5	None	0/5	None
**−/**−****						
Peripancreatic fat	0/5	None	0/5	None	0/5	None
Acinar	0/5	None	0/5	None	0/5	None
Endocrine	0/5	None	0/5	None	0/5	None

E.4 = embryonic age. p.i.* = postinfection. +/−** dam virus-infected, pup mock-infected. −/+ dam mock-infected, pup virus-infected. +/+ dam virus-infected, pup virus-infected. −/− dam mock-infected, pup mock-infected. None = no infiltration.

**Table 4.** Histopathology of pancreas tissues of challenged pups of dams infected in the second week of gestation (Day 10/E.10).

Pancreatic tissue of pups	Infiltration status in the pancreas at different days post-CVB4-E2 or mock infection					
	Number of positives/ total	Grade	Number of positives/total	Grade	Number of positives/ total	Grade
	Day 5 p.i.*		Day 12 p.i.		Day 21 p.i.	
+/+**						
Peripancreatic fat	0/5	None	1/5	+2	0/5	None
Acinar	3/5	+1 to +2	2/5	+2 to +4	5/5	+ 3 to +4
Endocrine	0/5	0	0/5	None	4/5	+1 to +2
**−/+**						
Peripancreatic fat	4/5	+1	1/5	+1	2/5	+1
Acinar	0/5	None	0/5	None	0/5	None
Endocrine	0/5	None	0/5	None	0/5	None
**+/−**						
Peripancreatic fat	5/5	+1 to +2	1/5	+1	3/5	+1
Acinar	0/5	None	0/5	None	0/5	None
Endocrine	0/5	None	0/5	None	0/5	None
**−/−**						
Peripancreatic fat	0/5	None	0/5	None	0/5	None
Acinar	0/5	None	0/5	None	0/5	None
Endocrine	0/5	None	0/5	None	0/5	None

E.10 = embryonic age. p.i.* = postinfection. +/−** dam virus-infected, pup mock-infected. −/+ dam mock-infected, pup virus-infected. +/+ dam virus-infected, pup virus-infected. −/− dam mock-infected, pup mock-infected. None = no infiltration.

**Table 5.** Histopathology of pancreas tissues of challenged pups of dams infected in the third week of gestation (day 17/E.17).

Pancreatic tissue of pups	Infiltration status in the pancreas at different days post-CVB4-E2 or mock infection					
	Number of positives/ total	Grade	Number of positives/total	Grade	Number of positives/ total	Grade
	Day 5 p.i.*		Day 12 p.i.		Day 21 p.i.	
+/+**						
Peripancreatic fat	3/5	+1 to +2	4/5	+1 to +4	3/5	+2 to +3
Acinar	2/5	+3 to +4	3/5	+3 to +4	5/5	+1 to +4
Endocrine	0/5	None	0/5	None	0/5	None
**−/+**						
Peripancreatic fat	4/5	+1 to +2	2/5	+1	0/5	None
Acinar	0/5	None	0/5	None	0/5	None
Endocrine	0/5	None	0/5	None	0/5	None
**+/−**						
Peripancreatic fat	3/5	0 to +1	1/5	+1	3/5	+1
Acinar	0/5	None	0/5	None	0/5	None
Endocrine	0/5	None	0/5	None	0/5	None
**−/−**						
Peripancreatic fat	0/5	None	0/5	None	0/5	None
Acinar	0/5	None	0/5	None	0/5	None
Endocrine	0/5	None	0/5	None	0/5	None

E.17 = embryonic age. p.i.* = postinfection. +/−** dam virus-infected, pup mock-infected. −/+ dam mock-infected, pup virus-infected. +/+ dam virus-infected, pup virus-infected. −/− dam mock-infected, pup mock-infected. None = no infiltration.

Pancreas tissues of the mock-infected control pups (−/−) were normal in all groups throughout the experiments. In pups (−/+) of dams mock-infected on day 4/E.4, the pancreas showed peripancreatic fat infiltration ranging from grade +1 (Figure 5(c)) to +3 (Figure 5(d)) at day 5 p.i. The infiltration subsided in this group of pups. On the other hand, pups of the other two virus-infected groups +/+ and +/– showed mild peripancreatic infiltration from day 12 p.i. to day 21 p.i. (grade +1 to +2) as shown in Table 3.

In the pancreas of pups +/+ of dams infected on day 10/E.10, the acinar tissues showed acute pancreatitis at day 5 p.i. (after these pups were challenged). Moreover, by day 21 p.i. in this group of pups, chronic pancreatitis was observed with infiltration of the islets. In the pups of dams infected on day 17/E.17, pancreatitis with pancreatic atrophy (grade +1 to +4 infiltration) was detected at day 12 p.i. (Figure 5(g,f)).

## Immunohistochemical (IHC) analysis

**Figure 6.**
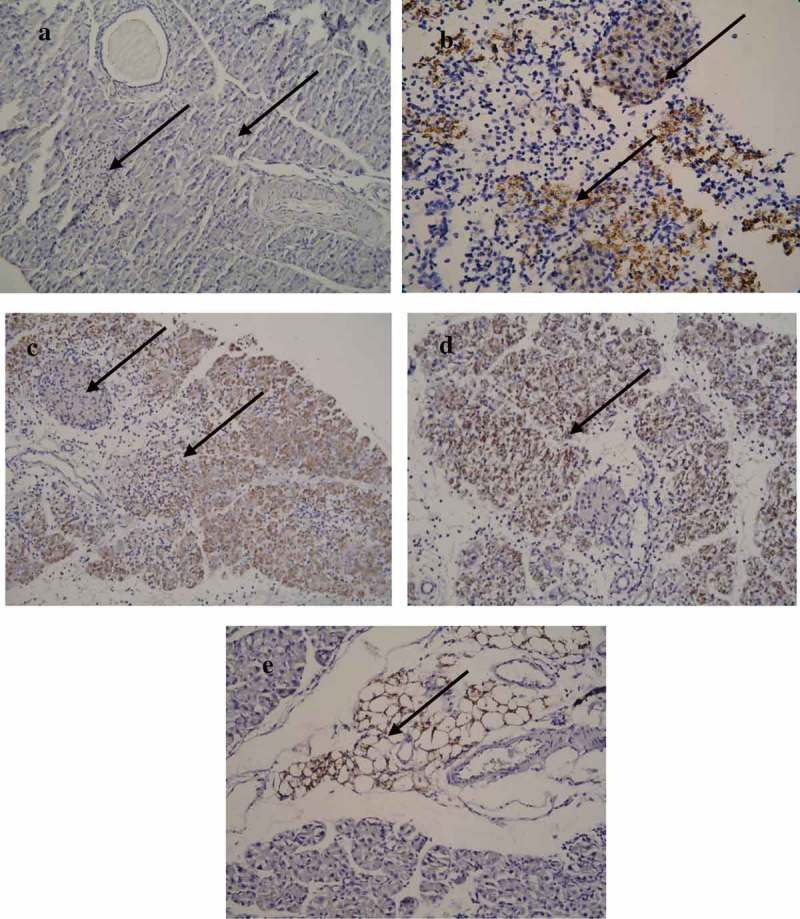
**Localization of VP1 in the pancreatic tissue**. (a) Absence of VP1 in the islet and exocrine tissue of a mock-infected pup of a dam mock infected on day 4/E.4 (−/−) (20×), pup sacrificed on day 5 p.i. (b) Presence of VP1 in the islet and exocrine tissue on day 3 p.i./E.20 of a dam infected with CVB4-E2 on day 17/E.17. (c) Presence of VP1 in the exocrine tissue but absence in the islet of virus challenged pup of a dam infected on day 10/E.10 (+/+), and of dam at day 17/E17. (d) Pups sacrificed on day 5 p.i. (20×). (e) Peripancreatic fat tissue showing VP1 positivity at day 5 p.i. in the pancreas of a virus challenged pup of a mock-infected dam (20×).

Localization of the virus by IHC analysis of viral protein (VP1) showed the presence of virus in the acinar tissue as well as the islets in the dams infected at day 17/E.17 of gestation as early as day 3 p.i./E.20. VP1 was observed in the exocrine pancreas and peripancreatic fat tissue in the pancreatic tissues of the pups of CVB4-E2-infected dams. However, some of the islets of the pups did not show VP1 (Figure 6(a–e)) while others showed mild to almost absence of positivity.

**Figure 7.**
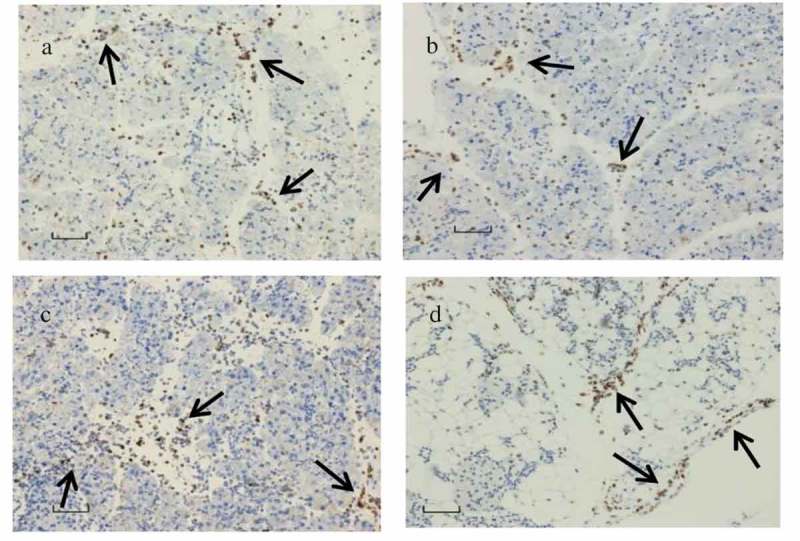
CD11b+ macrophage and LY6G+ neutrophils localization. (a) CD11b+ macrophage and (c) LY6G+ neutrophils in abundance seen in the pancreas tissue infected with CVB4-E2 on day 4 (E.4), collected on day 3 p.i. from a dam; (b) CD11b+ macrophages observed in the pancreas tissue collected on day 5 p.i. of a challenged pup of dam infected with CVB4-E2 on day 4 (E.4) whereas (d) shows CD11b+ macrophages seen in the pancreas tissue collected on day 21 p.i. of a challenged pup of dam infected with CVB4-E2 on day 17 (E.17).

Immunohistological staining was done for analyzing infiltration of CD11b+ and Ly6G+ cells in the pancreas of the dams and their pups. CD11b+ (Figure 7(a,b,d)) infiltration was present in both the dams and the pups at acute and late (day 21 p.i.) phases of infection. Presence of Ly6G+ (Figure 7(c)) was identified in the pancreas of the dams. However, Ly6G+ cells were almost absent in the infiltration at the acute and late phases of infection.

## Discussion

The most significant outcome of our study is that the infection of dams with CVB4-E2 during the second week of gravidity (day 10/E.10) and the challenge of their offsprings with the homologous virus leads to the affliction of the endocrine pancreas. In addition, the types of infiltrating cells between the infected dams and their challenged pups differed.

We have demonstrated that virus administered orally during the second week of gestation (day 10/E.10) caused pancreatitis which was severe on day 3 p.i./E.13 and increased to an acute inflammatory phase by day 5 p.i./E.15 showing infiltration in the exocrine tissue as well as in the islets (endocrine). Remarkably, the pups +/+ of these dams showed chronic pancreatitis and pancreatic atrophy with infiltration in the islets by day 21 p.i., although some pups from other groups such as the progeny (−/+) and pups of dams infected on day 4/E.4 and day 17/E.17 showed the presence of viral RNA at day 12 and day 21 p.i. and higher virus load. Presence of VP1 was mild or absent at day 21 p.i. in these tissues. We have shown that the islets were affected at later days p.i. in both the mother and the challenged offspring. We consequently suggest that islet infiltration or the chronicity was not related to (1) presence of viral RNA, (2) the number of copies of viral RNA, and (3) presence of VP1 in the islets but is influenced by the time of infection of the gestating mice showing that factors other than the virus can potentially attract the infiltrating cells into the islets in these mice. The other groups of dams as seen in Table 2 and offsprings (Tables 3–5) showed pancreatitis in the acinar tissue, but the islet infiltration was absent.

Different groups [26–40] have reported adverse outcomes on the course of gravidity after infection in the first or second weeks of gestation and even third week [26]. Some authors [28,29,35] attributed the adverse effects to the susceptibility of the fetus to the virus resulting in infection. Other researchers [27] suggest virus-induced pancreatic exocrine insufficiency in the mothers (dams) accompanied by a protein deficiency and hepatic-cell vacuolation. Whereas other groups [34,37] associate the damaging effect on the gravidity to the presence of virus in reservoirs such as the placenta and other tissues leading to the infection of the embryos. Expression of coxsackie-adenovirus receptors in the placenta, uterus, and dominantly in the embryonic brain and cardiac cells have been described [38,39], and possible physiological changes in the hormone levels were suggested by others [32]. In the present study, replicating virus was present in the umbilical cords and placentas by three passages. Our results suggest that the passage of virus transmission via placenta is possible. We contemplate that the amount of virus transmitted may differ from individual to individual because all placentas were not positive at day 5 p.i. This difference can be explained, as a variation due to the outbred immunocompetent (CD1) mouse strain used by us and not an inbred strain. Here, we need to mention that this part of our study is limited, and we have pooled pieces of umbilical cords/dam and placentas/dam at a single time point for the preparation of the organ suspensions.

Infection of gravid mice by different viruses has been discussed and reviewed [41]. This review underlines the necessity to explore disease mechanisms caused by viruses which have a potential to cause fetal morbidity and mortality. The authors have focused on human cytomegalovirus, rubella virus, human immunodeficiency virus, and herpes zoster virus. It appears that the viruses which have a direct cytopathic effect (CPE) depending on its life cycle may have a direct effect on the developing placenta and fetus. On the other hand, the viruses that induce a shift in the immune profile from anti-inflammatory to proinflammatory induction of cytokines may affect the developing fetus and its immune system. Futhermore in case of rubella and other viruses such as coxsackieviruses, neonatal T cells react with viral peptides and cross-react with an antigen in the insulin-producing pancreatic beta islet cells resulting in an autoimmune destruction of the islet cells. We therefore studied the basic differences in the infiltrating cells of the pancreas. Infiltrating cells, CD11b+ and Ly6G+, were analyzed. Our immunohistological staining results of the pancreas of the dams and their pups showed the pronounced presence of Ly6G+ only in the dams, whereas CD11b+ infiltration was present in both the dams and the pups at different intervals. The first direct implication of this finding is that there is a difference between the immune responses to the infection in the above two groups. We may therefore predict a difference in the production of cytokines and downstream inflammatory reactions. Ly6G protein is limited to neutrophils [42]. Monocytes, macrophages, lymphocytes, and plasmacytoid dendritic cells (DCs) do not possess this protein. Ly6G expression is high in cells recruited to inflamed sites. Furthermore, CD11b+Ly6C+Ly6G- and CD11b+Ly6C+Ly6G+ can migrate to the site of virus infection [42]. These cells are known to initiate different functions that control virus replication, prevent systemic spread of virus, but also reduce tissue damage. Presence of CD11b+ was suggested to show an early-onset infection or suspected infection in infants at risk or recent infections [43]. Rechallenge of immunized mice with viruses lymphocytic choriomeningitis virus and vesicular stomatitis virus, after maternal respiratory syncytial virus infection, has been shown to alter postnatal offspring immunity [44]. The Ly6C+Ly6G+ cells that infiltrate into the site can mediate both effector (Type I interferon production) and immunomodulatory (reduction of tissue damage) properties in response to virus infection [42]. Presence of these cells is important for the protection of tissue from further damage mediated by the inflammatory response. Besides the macrophages, neutrophils are also known to be involved in the inflammatory responses induced by a number of viral infections [45,46]. During herpes simplex virus infections, neutrophils have been shown to play a clear role in limiting virus replication [47–49]. Taking into consideration the published literature and our own results, we may contemplate that the presence of the Ly6G+ cells in the infected dams plays a role in the virus clearance and tissue recovery in the dams which is a typical reaction to virus-infection. Whereas the atypical absence of these Ly6G+ cells in the pancreas of infected offsprings is peculiar and maybe an influence of the infection during pregnancy may predict a slow viral or antigen clearance and future tissue damage. Although this may explain the chronicity and severe pancreatitis in the progeny, we will have to investigate further why the neutrophils are absent.

Accumulation of interferon (IFN)-gamma secreting T cells within the central nervous system (CNS) was suggested to limit Ly6G+ neutrophil infiltration during the mouse hepatitis virus -JHM strain infection through inhibition of macrophage inflammatory protein (MIP)-2 expression [50]. The absence of Ly6G+ in the infiltration of the challenged pups and observation of chronic atrophy in the acinar tissue point to an imbalance in the offspring-immune system which may lead to autoimmunity by tipping the balance between innate and adaptive immune responses. Alternatively, it is possible that the occurrence of chronic atrophic acinar tissue in the absence of inflammation may result from events independent of immune mechanisms. Investigations into these aspects may shed new insights into the development of acinar pathologies in enteroviral infections.

The mechanism of *in utero* transmission of viruses depends on the infecting virus. We have to also consider that the development and formation of the pancreas in rodents starts at E.8.0–10.0 immediately after closure of the anterior endoderm. A thickening of the endoderm (bud) appears dorsally with another bud is formed on the ventral side (called as dorsal and ventral endoderms). The two buds develop in opposite directions and fuse to form the pancreas. Formation of islets and expression of several antigens takes place, such as pancreatic and duodenal homeobox (Pdx1), motor neuron and pancreas homeobox 1 (Hlxb9), pancreas associated transcription factor 1a (Ptf1a), homeobox protein Nkx-6.1 (Nkx6-1), and homeobox protein Nkx-2.2 (Nkx2-2) in the common pancreatic progenitor cells in the epithelium with homeobox protein Nkx-6.2 (Nkx6-2) and SRY (sex-determining region Y)-box 9 protein (Sox9) starts also at this period of the embyonic development [51,52]. We had hypothesized possible mechanisms which could induce pancreatic pathological changes in the offsprings (+/+) of infected dams [26]. In this study, we show that (a) histopathological changes were independent of the following: the amount or presence of viral RNA; amount of viral RNA copies and presence of the VP1 in the tissue; (b) the mechanism of tissue necrosis and infiltration may result from altered innate immune mechanism of the challenged pups of the infected dams and this response differs from the maternal innate immune response and not the enhanced adaptive immunity. Taken together, our data may also point to a possibility that the pathological changes in the pancreas occur primarily due to viral damage with the first initial exposure to the virus or that viral antigens are likely to persist in those affected cells. To this end, it may be worth investigating to determine the transcriptome profiles in healthy vs. diseased tissue with an expectation that the differentially expressed genes may in fact determine the pathological outcomes. We plan to pursue these aspects in the future studies.

Our model and data are in line with the proposition suggested by other authors [22–25,53,54] that there is an association between maternal enteroviral infection during pregnancy and T1D in their children in the seroepidemiological studies.

In conclusion, our model shows that CVB4-E2 infection during gravidity influences the histopathology of the pancreas of both infected dams and their challenged pups. The infection leads to chronic changes in the pancreas at late stages (days p.i.). We also suggest that more attention should be directed toward enteroviral infections during pregnancy which may have consequences on the health of the offspring.

## Material and methods

### Virus and cells

Coxsackievirus B4-E2 was kindly provided by Prof. J. Galama (Department of Microbiology, Radboud University, Nijmegen, the Netherlands) and Prof. M. Roivainen (Enterovirus Laboratory, Department of Virology, National Public Health Institute, Helsinki, Finland), with permission from Prof. J. W. Yoon (Department of Microbiology and Infectious Diseases, Julia McFarlane Diabetes Research Centre, Faculty of Medicine, University of Calgary, Alberta, Canada). The virus was propagated in Green monkey kidney (VERO) cells. Human epidermoid carcinomas of the larynx (Hep-2) cells (Office of Public Health, Bratislava, Slovak Republic) were used for virus isolations from organs. These cells are more sensitive to primary isolation from the mouse organs. Cells were grown in Eagle’s Minimum Essential Medium (EMEM) and supplemented with 5–10% heat-inactivated fetal bovine serum for cell growth or 2% for cell maintenance and infection, 100 U/ml penicillin (PNC), 100 µg/ml streptomycin (STM), and 1% HEPES (4-(2-hydroxyethyl)-1-piperazineethanesulfonic acid ).

### Mice

The experimental mouse study and protocols were reviewed and approved by the Institutional Animal Ethics Committee of the Slovak Medical University followed by the State Veterinary and Food Control Authority of the Slovak Republic (dated 22 March 2007, number C.k Ro 3035/07-221/3). All rules and regulations were followed according to the requirements of the Animal Ethics Procedures and Guidelines of the Slovak Republic.

The mice were maintained in the specific pathogen-free Laboratory Animal Facility of the Slovak Medical University in Bratislava. All mice had free access to food and water and were kept in rooms controlled for temperature (22 ± 0.5°C), automatic light, reverse 12/12 h light/dark cycle, and humidity (55% ± 5%).

CD-1 outbred male and female mice (8 weeks old) weighing about 25 g were obtained from Faculty of Medicine Masaryk University, Brno, Czech Republic. Mice were caged with sterile bedding, water, and commercial food pellets (Topdovo, Trnava, Slovak Republic).

### Infection during gestation

After the quarantine period of 10 days, one male mouse per 3 female mice was placed in a single cage for planned gestation. The female mice were checked daily. Increase in weight of the female mice was noted, and vaginal plugs were checked to estimate the exact time of gestation.

#### Infection of dams

Mice were infected at three different time points: days 4/E.4 (first week), 10/E.10 (second week), and 17/E.17 (third week) of gestation. Figure 1 shows the schematic diagram of the gestational period of mice with the E age, time points at which the dams were infected with the virus, and the days p.i. when these mice were dissected.

The gravid dams were inoculated either with 0.2 ml of CVB4-E2 at a dose of 1 × 10^5.5^ TCID_50_ or with 0.2 ml of phosphate-buffered saline (PBS) by oral route as described by Bopegamage et al. [35].

#### Infection of pups

Pups were separated from their mothers 3 weeks after birth (natural time for weaning) and put into separate cages. To reduce the effects of gender difference, only male pups were used for the present study (the female pups were kept for another study). The pups were challenged with 0.2 ml of 10^5.5^ TCID_50_ of CVB4-E2 or PBS, 4 days after weaning (25 days after birth).

#### Litter groups

Litter of the dams infected on day 4/E.4 (first week) of gravidity were challenged with CVB4-E2 (+/+); litter of the dams infected with virus on day 4/E.4 (first week) were challenged with PBS (±); litter of the dams mock-infected on day 4/E.4 (first week) were challenged with CVB4-E2 (−/+); litter of the dams mock-infected on day 4/E.4 (first week) of gravidity were challenged with PBS (−/−). Similar combinations of groups were formed with the litter of the dams infected on day 10/E.10 (second week) and day 17/E.17 (third week).

## Organ collection from infected mice

Infected dams were sacrificed on different days p.i. (Figure 1). Dams orally infected in the first week of gravidity were dissected with four mice per time interval, on days 3, 5, and 14 p.i. Similarly, gravid mice infected in the second week of gestation were dissected (four per time interval) on days 3 and 5 p.i., and four dams from the last group infected in the third week of gravidity were dissected on day 3 p.i. Control (mock-infected) mice were parallelly sacrificed. Blood, heart, pancreas (dams and pups), placenta, and umbilical cords were collected (from dams). Placenta and umbilical cords were each pooled/dam due to small sample size.

CVB4-E2 challenged pups and the mock-infected control pups (five mice from each group) were sacrificed at selected time points, on days 5, 12, and 21 p.i.; blood, heart, and pancreas were collected as described previously [53].

## Evaluation of infectious virus in organ suspensions

Organ suspensions from the snap-frozen tissues were prepared, and blind passages were performed as described previously [55,56]. The resulting supernatants were collected, and antibiotics (200 U/ml PNC and 200 μg/ml STM) were added. The organ suspensions were frozen at −80°C and used for blind passages in tissue cultures. We did not titrate the virus in the organs but made blind passages. The absence of CPE in the first passage does not mean the absence of replicating virus.

Twenty-four-hour monolayers of Hep-2 cell line grown in single-use tissue culture flasks (50 ml) were infected with 0.5 ml of the original organ suspensions. After 45-min incubation at 37°C, medium EMEM was supplemented with 2% fetal bovine serum and 1% HEPES, and antibiotics (PNC 100 U/ml, STM 100 μg/ml) was added. Cultures were incubated at 37°C and CPE was checked till day 5 p.i. If CPE was observed, the flasks were freeze-thawed two times and suspensions were centrifuged at 1000 g, 4°C, for 20 min (Minifuge T, Heraeus Sepatech). The same procedure was followed if CPE (blind passages) was not observed at day 5 p.i. Supernatants were used for further infection of cell monolayers. Three blind passages were carried out with organ suspensions of the infected mice; organs of the control mice were passaged only once. All passages of the infected organs were stored at −80°C.

## RNA extraction

RNA extraction from snap frozen organs has been described in our previous study [55] using the PureLink® RNA Mini Kit according to the manufacturer (Invitrogen, USA).

## Reverse-transcription polymerase chain reaction (RT-PCR) and nested PCR analysis

RT-PCR and nested PCR have been described previously [35,37,38]. PCRs were performed in T100^TM^ Thermal Cycler (Bio-Rad). Both cDNA synthesis and cDNA amplification were performed in a single tube by using the SuperScript^TM^ III One-Step RT-PCR System with Platinum®*Taq* High Fidelity (Invitrogen). For the nested reaction, Platinum® PCR SuperMix (Invitrogen) was used. The primers directed to the highly conserved sequences in the 5´untranslated region of the EV genome were obtained from Microsynth (Germany). Positive and negative RNA control, beta-actin internal controls, DNA control, and water blank controls were included in each run.

The PCR products were separated on a 2% agarose gel stained with ethidium bromide, at 150 V, 400 mA for 1.5 h, visualized, and documented by UV illumination in Gel Documentation XR+ Imaging System (Bio-Rad).

## Real-time reverse transcriptase-PCR (real-time RT-PCR)

Quantification of the isolated RNA was performed by using the EliGene® Enterovirus LC Kit (Elisabeth Pharmacon) according to the manufacturer’s instructions. RNA was reverse-transcribed at 55°C for 15 min followed by one cycle of denaturation at 95°C for 5 min. PCR amplification was carried out for 50 cycles at 95°C for 15 s, at 55°C for 30 s, and at 72°C for 25 s. The last step was followed by one cycle at 40°C for 1 min. Real-time RT-PCR was performed on the CFX96^TM^ Real-Time system (Bio-Rad).

## Histolopathological analysis

The tissues section of the pancreas and heart were examined for evidence of inflammatory, necrotic, and morphological changes. Serial 4–7-µm-thick sections of formalin fixed paraffin embedded heart and pancreas tissues were stained with haematoxylin-eosin and graded (1–4) for infiltration and necrosis as described previously [55,56].

## IHC analysis

Viral protein was localized in the pancreas of dams and the pups by using primary antibody with an epitope for the VP1 (Monoclonal Mouse Anti Enterovirus Clone 5-D8/1, Dako; dilution 1:250). Other reagents used were the secondary antibody included in the Dako ARK (Animal Research Kit) and peroxidase for mouse secondary antibodies (Dako). The methodological details are described in our previous studies [57,58].

Pancreata were examined for the presence of neutrophils and macrophages by IHC. Rat anti-mouse Ly6G (clone, 1A8, 1:250; Leinco), rabbit anti-mouse CD11b (clone, EPR1344, 1:3500; Abcam), and their corresponding isotype controls were used to detect neutrophils and macrophages, respectively. In brief, after deparaffinization, rehydration, and blockade of endogenous peroxidase activity with 3% hydrogen peroxide, antigen retrieval was performed by treating the sections with 10 mM sodium citrate buffer (pH 6) in a pressure cooker. Sections were then blocked for 30 min with 5% nonfat dry milk and incubated overnight with primary antibodies at 4°C, followed by incubation with secondary antibodies, namely, donkey anti-rat IgG or goat anti-rabbit IgG conjugated with horse radish peroxidase (Vector Laboratories, Burlingame, CA; and Abcam) for 2 h at room temperature. Diaminobenzoic acid was added as a substrate for color development, and sections were counterstained with hematoxylin.
